# Identification and Expression Analysis of PIN-Like (PILS) Gene Family of Rice Treated with Auxin and Cytokinin

**DOI:** 10.3390/genes6030622

**Published:** 2015-07-16

**Authors:** Tapan Kumar Mohanta, Nibedita Mohanta, Hanhong Bae

**Affiliations:** 1 Department Biotechnology, Biotechnology Building, Techno Park, Yeungnam University, Daehak Gyeongsan 712749, Korea; 2 Department of Biotechnology, North Orissa University, Takatpur, Baripada, Mayurbhanj, Orissa 757003, India; E-Mail: nibu.biotech@gmail.com

**Keywords:** OsPIN, AtPIN, OsPILS, AtPILS, transmembrane domain, auxin, hydrophilic domain, auxin efflux carrier

## Abstract

The phytohormone auxin is one of the most important signaling molecules that undergo accumulation or depletion in a temporal or spatial manner due to wide arrays of changes in developmental or stress programs. Proper distribution, maintenance and homeostasis of auxin molecules across the plant systems are one of the most important phenomena required for proper growth and development of plant. The distribution and homeostasis of auxin is maintained by auxin transport systems across the plant. The auxin transportation is carried out by auxin transporter family proteins, popularly known as auxin efflux carriers (PINs). In this study, a sub-family of auxin efflux carrier (*OsPILS*) genes was identified from *Oryza sativa* and relative expression profile was studied by treating them with auxin and cytokinin. *Oryza sativa* encodes seven putative sub-cellularly localized transmembrane OsPILS genes distributed in five chromosomes. Differential expression of *OsPILS* genes was found to be modulated by auxin and cytokinin treatment. In auxin treated plants, all *OsPILS* genes were up-regulated in leaves and down regulated in roots during the third week time period of developmental stages. In the cytokinin treated plants, the maximum of *OsPILS* genes were up-regulated during the third week time period in root and leaf tissue. Regulation of gene expression of *OsPILS* genes by auxin and cytokinin during the third week time period revealed its important role in plant growth and development.

## 1. Introduction

The phytohormone auxin plays important roles in plant growth and development by modulating processes such as cell division, cell elongation, vascular differentiation, apical dominance, tropic growth, embryogenesis, cell polarity, root architecture and root organogenesis [1,2,3,4,5]. The auxins synthesized in the aerial parts of the plant are transported to root tips via the vascular system and polarized auxin distribution system, where they maintain auxin homeostasis and distribution [6,7]. In addition to the vascular transport system present in plants, a cell to cell transport system that covers long and short distance auxin distribution is also present in the tissue [8]. The polar auxin efflux carrier (PIN) gene plays important roles in auxin distribution and homeostasis in plants in a polarized manner [2,9,10]. The *AtPIN1*gene was the first PIN gene to be cloned from the plant *Arabidopsis thaliana* [11]. When it was reported that an *Arabidopsis* pin-formed1 (pin1) with an auxin transport defective mutant develops pin-like inflorescence, it became clear that PIN protein plays a significant role in auxin efflux from cells [11]. Twelve PIN genes have been found in rice and eight PIN genes in *Arabidopsis thaliana* [12,13]. The PIN gene shows distinct patterns of cellular and sub-cellular localization in roots and shoots [12,14,15]. The AtPIN1 localizes polarly in the plasma membrane, and upon pharmacological disruption, it immediately relocalizes, suggesting that the conceptual basis of auxin flux affects tropic response and patterning [2,7,16,17]. It has been reported that the rice *OsPIN1* gene is expressed in root caps, *OsPIN1b*, *OsPIN1c* and *OsPIN9* are predominantly expressed in the stele, and *OsPIN1b*, *OsPIN1c*, *OsPIN5a* and *OsPIN5b* are expressed in meristem [13].

The plant specific PIN gene family of auxin efflux carriers consists of integral membrane proteins that contain an inner and outer transmembrane domain and central hydrophilic domain [18,19,20]. The N-terminal and C-terminal regions of PIN proteins are conserved and the central hydrophilic loop region is dynamic in nature among different PIN proteins [21,22,23]. Based on the divergence of the central hydrophilic loop, PIN proteins are divided into different groups [19]. Although several PIN genes from different species and their function have been reported to date, only a few reports are available regarding the role of PIN like (PILS) genes in plants [10]; therefore, we attempted to analyze the role of *OsPILS* genes by treating them with auxin and cytokinin.

## 2. Materials and Methods

### 2.1. Bioinformatics Analysis

The PIN likes (PILS) gene family of *Oryza sativa* was identified from publicly available rice genome database (www.rice.plantbiology.msu.edu) [24]. The *OsPILS* genes identified from *Oryza sativa* genome were named according to the orthology based nomenclature of *Arabidopsis thaliana AtPILS* genes [25]. The TMHMM (prediction of transmembrane helices in protein) server (http://www.cbs.dtu.dk/services/TMHMM/) was used to analyze the transmembrane domain structure of OsPILS proteins. The Swiss model work space (http://swissmodel.expasy.org/workspace/) was used to predict the auxin efflux carrier domain of OsPILS proteins. The multiple sequence alignment of OsPILS proteins with orthologous AtPILS proteins of *Arabidopsis thaliana* was carried out using clustalw software and protein weight matrix programme used was BLOSUM. The phylogenetic tree of OsPILS, AtPILS, OsPIN and AtPIN of *Arabidopsis thaliana* and *Oryza sativa* was constructed using MEGA5 software [26]. To create the phylogenetic tree, the protein sequences of AtPILS and OsPILS were subjected to clustalw programme to generate a clustal file. The resulted clustal file was then converted to MEGA file format by MEGA5 software. The resulted MEGA file of PILS was run in MEGA5 software to construct the phylogenetic tree. Different statistical parameters used to construct the phylogenetic tree were: statistical method, maximum likelihood; test of phylogeny, bootstrap method; no. of bootstrap replication, 1000; substitution type, amino acids; models/methods, Jones-Taylor-Thornton (JTT) and branch swap filter was very strong. Sub-cellular localization of OsPILS proteins was predicted using online available software CELLO v.2.5: sub-cellular localization predictor [27].

### 2.2. Plant Materials and Growth Conditions

The *Oryza sativa* L. indica cultivar group var Pusa Basmati 1 were grown in half MS (Murashige and Skoog) (half strength of MS basal agar medium) agar media in sterile glass bottle supplemented with 5 µM of auxin [18,28,29] and cytokinin [30]. The plants were then grown up to four weeks at 16/8 h day light period at 28 °C. The light intensity for growth of rice plant was kept in 700 µmolm^−2^s^−1^. Phytohormone treated plants were harvested at seven days, 14 days, 21 days and 28 days (weekly intervals), after which the harvested plants were immediately transferred to liquid nitrogen and preserved them for further analysis. Three biological replicate samples were used for this study. Total RNA was extracted from leaf and root samples collected from treated and non-treated plants. The isolated RNA was the subjected to cDNA synthesis using Fermentas RevertAid first strand cDNA synthesis kit according to manufacturer instructions. Briefly, reactions were prepared by adding 1.5 µg total RNA, 2 µL of 10× RT buffer, 2 µL of 10 mM dNTPs mix, 2 µL of random primers, 1 µL of reverse transcriptase, 1 µL ribolock RNase inhibitor and nuclease free sterile water up to 20 µL. The reaction mixtures were then subjected to thermal incubation at 42°C for 60 min followed by reaction termination at 70 °C for 5 min.

### 2.3. qRT-PCR Analysis for Gene Expression

Primers specific for the *OsPILS* genes were designed using the primer3 (http://primer3.ut.ee/) software. A detailed list of primers is presented in Table 1. Proper care was taken to design the primers for each gene so that there was no overlapping amplification among different *OsPILS* genes. As the central hydrophilic region of OsPILS proteins is highly dynamic in nature, it provided a useful platform for designing specific primer sets. The specificity of each *OsPILS* amplicon was further confirmed by gene sequencing. Quantitative real time PCR of *OsPILS* genes was conducted using Applied Biosystems^®^ viiA^TM^ 7 real time PCR system (Foster City, CA, USA) with SYBR green PCR master mix (2×) (Fermentas, Foster City, CA, USA) and ROX as a passive reference standard to normalize the SYBR green fluorescent signal. The reaction mixture consisted of 1 µL of cDNA as template (1:10 dilution of cDNA from 20 µL of the RT-PCR product), 12.5 µL SYBR green qPCR master mix (2×), 1 µL of each forward and reverse primer and nuclease free water up to 25 µL. The thermal profile of the qRT-PCR reaction was as follows: initial polymerase activation for 10 min at 95 °C, followed by 40 cycles of 30 s at 95 °C, 30 s at 60 °C, and 30 s at 72°C. Triplicate biological samples were used for qRT-PCR analysis. Reference housekeeping genes actin was used to calibrate and normalize the result of control (untreated) and treated samples for real time PCR analysis. The relative expression of OsPILS genes were calculated using 2^−Δ∆CT^ method [31].

**Table 1:** List of primers used for analysis of relative expression of OsPILS genes.

Gene Name	Locus ID	Forward Primer	Reverse Primer
OsPILS1	LOC_Os09g31478.1	AGCAAGCGTCCAGCCTTCAG	CTCTCCCCCACGCCGAACAG
OsPILS2	LOC_Os08g09190.1	CTCCAGCCACCAACCATCG	CGGGAGAATCAGGAGCCTTGC
OsPILS5	LOC_Os07g20510.1	GTCGTCCTCGCCTCCATCCA	CGCACCTTCCAAAGCAGAGC
OsPILS6a	LOC_Os01g60230.1	GATGGAGAGGTCGCTGATGGAG	GCGAGAACACAAGCCCATTG
OsPILS6b	LOC_Os05g40330.1	GAGGTCGGTGCTGGAGATGGTG	GGCAAGGAAGGAGCAGAGAGAACAC
OsPILS7a	LOC_Os09g38130.1	CCGCTCTCGTGGGCTCTCCT	GCCGCCTCCTCATCAGCGTA
OsPILS7b	LOC_Os09g38210.1	GAAGATGGCAACCCGTTCGG	CCTCCTCATCGGCGTAAGCA

## 3. Results

### 3.1. Identification and Bioinformatics Analysis of OsPILS Genes

**Figure 1:**
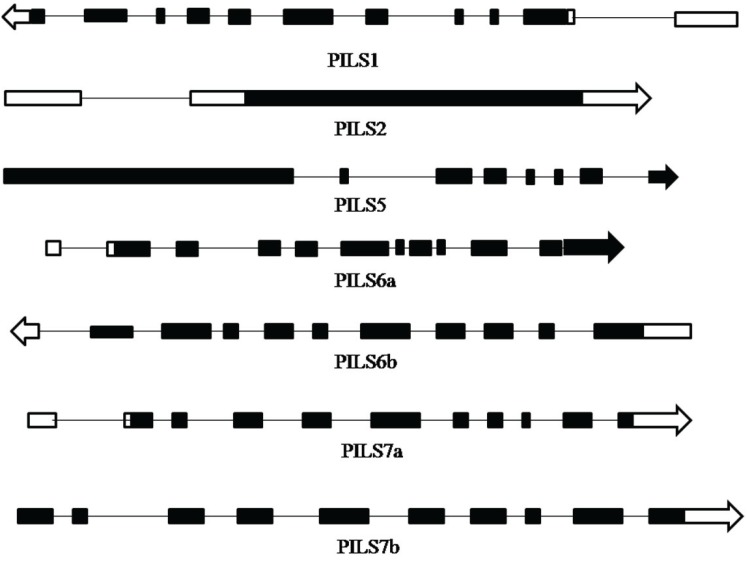
Genomic organization of different OsPILS genes. The black colored boxes represent the exons and lines represent the introns of different OsPILS genes. In the majority of cases, the majority of OsPILS genes contain 10 exons.

**Figure 2:**
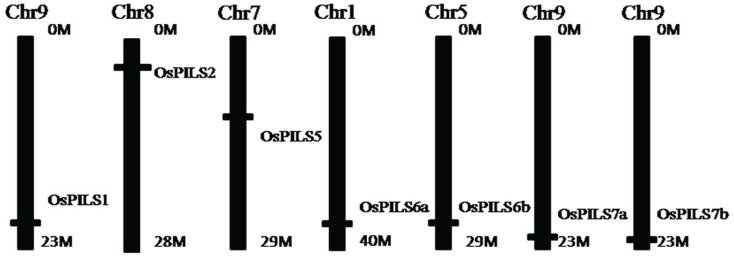
Distribution of OsPILS genes in rice chromosomes. All seven OsPILS genes are distributed toward the distal end of the chromosome. Chromosome 9 harbors three OsPILS genes, whereas chromosomes 1, 5, 7 and 8 harbor one OsPILS gene each.

A total of seven rice *OsPILS* (PIN likes) genes were identified from the rice genome annotation database (Table 2) [24,32]. The *OsPILS1* was observed to be the smallest and *OsPILS5* was the largest gene, with open reading frames (ORFs) of 1242 and 3840 bp, respectively (Table 2). The genomic organization of *OsPILS* genes reveals that *OsPILS1*, *OsPILS6a*, *OsPILS6b*, and *OsPILS7a* contain 10 introns each, while *OsPILS5* has seven, *OsPILS7b* nine and *OsPILS2* have only one (Figure 1). The chromosomal distributions of *OsPILS* genes show that *OsPILS1*, *OsPILS7a* and *OsPILS7b* are distributed in the distal end of chromosome 9, whereas *OsPILS2* is present in chromosome 8, *OsPILS5* in chromosome 7, *OsPILS6a* in chromosome 1 and *OsPILS6b* in chromosome 5 (Figure 2).

**Table 2:** The genomic information of OsPILS genes. The OsPILS5 gene has the longest ORF of 3840 nucleotides length. Among seven OsPILS genes, four of them contain 10 introns in their gene. The OsPILS2 and OsPILS5 resides in acidic isoelectric point (pI) range and all other OsPILS are resides in basic pI range.

Gene Name	Locus ID	ORF	No. of Amino Acids	No. of Introns	5'–3' Coordinates	pI
OsPILS1	LOC_Os09g31478.1	1242	414	10	Chr9: 18983481–18978136	8.38
OsPILS2	LOC_Os08g09190.1	1368	456	1	Chr8: 5322709–5325262	6.93
OsPILS5	LOC_Os07g20510.1	3840	1280	7	Chr7: 11845862–11851637	6.91
OsPILS6a	LOC_Os01g60230.1	1299	433	10	Chr1: 34834581–34838747	8.02
OsPILS6b	LOC_Os05g40330.1	1314	438	10	Chr5: 23700670–23697738	8.20
OsPILS7a	LOC_Os09g38130.1	1287	429	10	Chr9: 21963498–21968222	7.02
OsPILS7b	LOC_Os09g38210.1	1272	424	9	Chr9: 22007511–22010336	7.23

**Figure 3:**
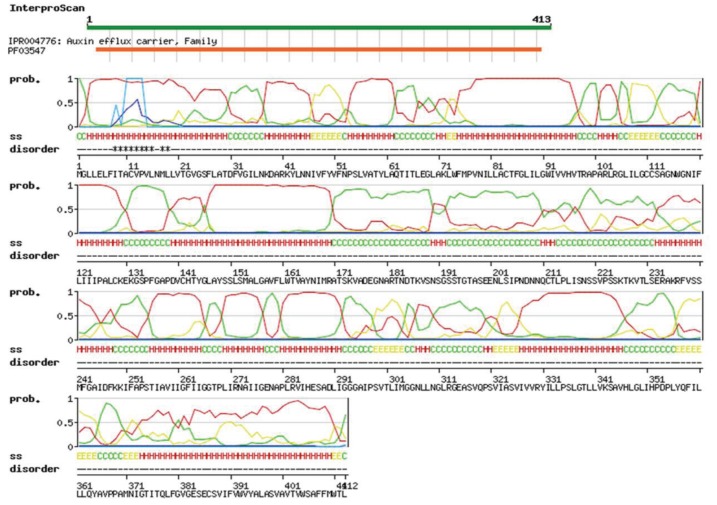
Auxin efflux carrier domain of the OsPILS gene. The Swiss workspace model (http://swissmodel.expasy.org/workspace/) was used to predict the auxin efflux carrier domain of OsPILS genes. All OsPILS genes contain auxin efflux carrier domain.

**Figure 4:**
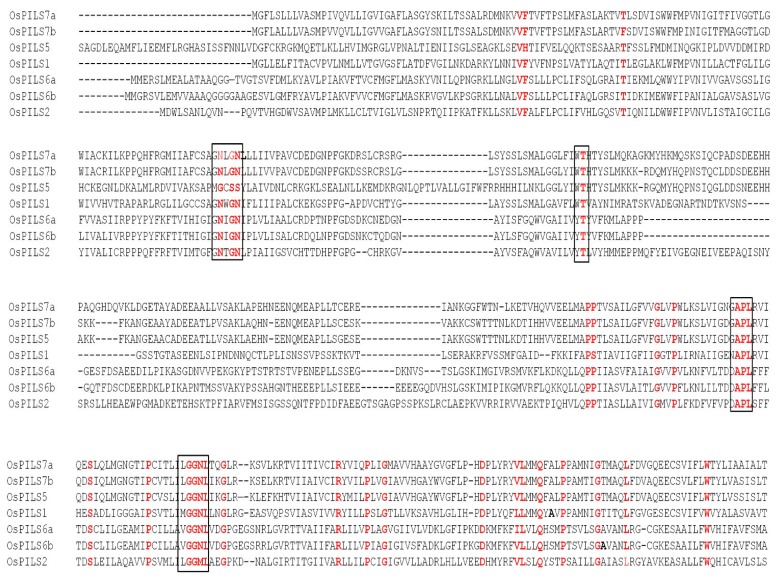
Multiple sequence alignment of OsPILS proteins. Alignment shows presence of several conserved amino acid residues and motifs in the N- and C-terminal region. The major conserved motif found in the N-terminal region is N-x-G-N (in box). The C-terminal region contains conserved A-P-L and G-G-N-L (in box) consensus sequences. Although the central hydrophilic region of the OsPIL protein is highly dynamic, it still contains conserved threonine (boxed) in the central hydrophilic region.

The rice OsPILS proteins were subjected to analysis using the Swiss workspace model to predict their auxin efflux carrier domains. The results revealed that all OsPILS proteins contains auxin carrier domain (Figure 3). Multiple sequence alignment of protein sequences of OsPILS was conducted to identify the conserved domains and motifs of OsPILS proteins. The sequence analysis revealed the presence of several conserved domains and motifs within the OsPILS protein sequence (Figure 3). These conserved sequences are present in both the N- and C-terminal region of the protein. The central hydrophilic region is highly variable in nature. The major N-terminal conserved domain found in the OsPILS proteins was N-x-G-N (except OsPILS5). Instead of containing an N-x-G-N consensus sequence, OsPILS5 contain a G-x-S-S consensus sequence, while the C-terminal region contains conserved A-P-L and G-G-N-L (Figure 4) consensus sequences. The central hydrophilic domains have no conserved domains, but contain conserved threonine amino acid in it. It was previously reported that the central hydrophilic region of PIN proteins does not have a conserved structure and are variable in nature [19,33]. However, in our study, we observed the presence of conserved amino acid in OsPILS. To better understand the sequence similarities of PIN proteins with OsPILS proteins, multiple sequence alignment of OsPILS, OsPIN, AtPILS and AtPIN proteins was conducted. The result revealed that no sequence similarity exists between PIN and PILS proteins (Supplementary data file 1). Additionally, sequence alignment showed that alignment of the OsPILS protein starts at the end of the alignment of PIN proteins (Supplementary data file 1). It has been reported that PILS genes are evolutionarily conserved among unicellular algae to higher eukaryotic plants such as gymnosperms and angiosperms [10]. The beginning of OsPILS proteins alignment at the end of the PIN protein alignment suggests that both *PINs* and *OsPILS* genes coexisted in the early period of evolution, then diversified and diverged as plants evolved from simpler aquatic habitats to complex terrestrial habitats.

**Figure 5:**
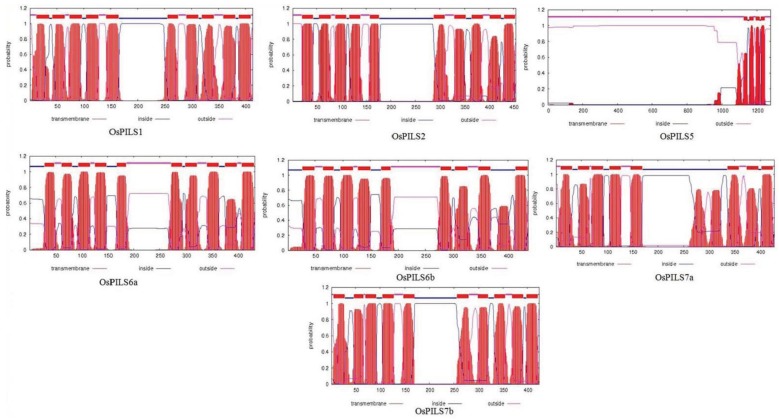
The prediction of transmembrane domains of OsPILS genes using TMHMM server 2.0). The prediction shows that maximum of OsPILS genes contained a short transmembrane domain.

The auxin efflux carrier proteins are membrane bound [34,35]; therefore, the protein sequences of OsPILS were analyzed to confirm whether they are membrane bound or not. All OsPILS proteins were observed to be transmembrane bound and present inside as well as outside of the membrane, except for OsPILS5, whose domain was found to be only present outside of the membrane (Figure 5).

### 3.2. Phylogenetic Analysis of OsPILS

**Figure 6:**
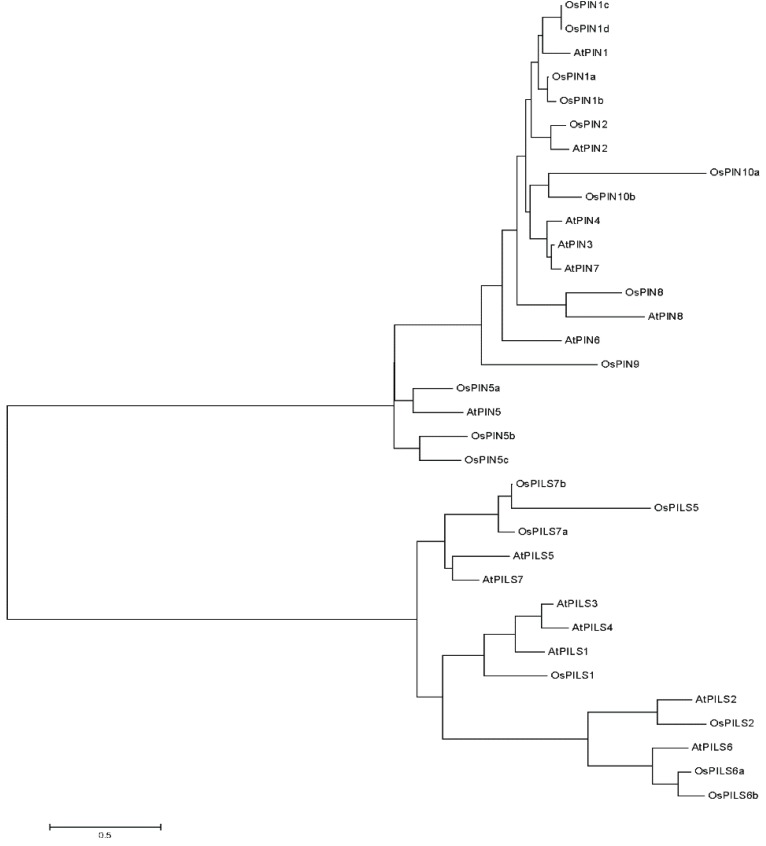
The phylogenetic tree of OsPIN, AtPIN, AtPILS and OsPILS genes. The phylogenetic analysis revealed PINs and PILS are grouped into two distinct clades. In the phylogenetic tree, the short transmembrane domain containing the OsPIN5 and AtPIN5 protein lies proximately towards the short transmembrane domain containing the AtPILS and OsPILS protein. These findings indicate that OsPILS proteins are short transmembrane domain containing proteins.

The phylogenetic analysis of OsPILS proteins with OsPIN, AtPILS and AtPINs protein was conducted using the MEGA5 software [26]. The result revealed that the PIN and PILS proteins fall into two different clades (Figure 6), while the OsPILS clades fell into three different sub-groups. In the phylogenetic tree, OsPILS5 is grouped with AtPILS5, AtPILS7, OsPILS7a and OsPILS7b whereas OsPILS1 is grouped with AtPILS1, AtPILS4, and AtPILS3. The OsPILS2 is grouped with AtPILS2, AtPILS6, OsPILS6a and OsPILS6b (Figure 6). The phylogenetic study clearly demonstrated the absence of OsPILS3 and OsPILS4 in rice, which are orthologous counterparts of the AtPILS3 and AtPILS4, respectively.

### 3.3. Expression of OsPILS Genes in Leaf Tissue Treated with IAA

To determine the expression pattern of *OsPILS* genes in rice, leaf tissues treated with 5 µM IAA (indole-3-acetic acid) were subjected to quantitative real time PCR (qRT-PCR) analysis. A small part of the open reading frame of the *OsPILS* genes was taken for qRT-PCR analysis. The expression of *OsPILS* genes from untreated rice plants was used as a control sample. All experimental analyses were carried out using seven, 14, 21 and 28 day old rice plants.

**Figure 7:**
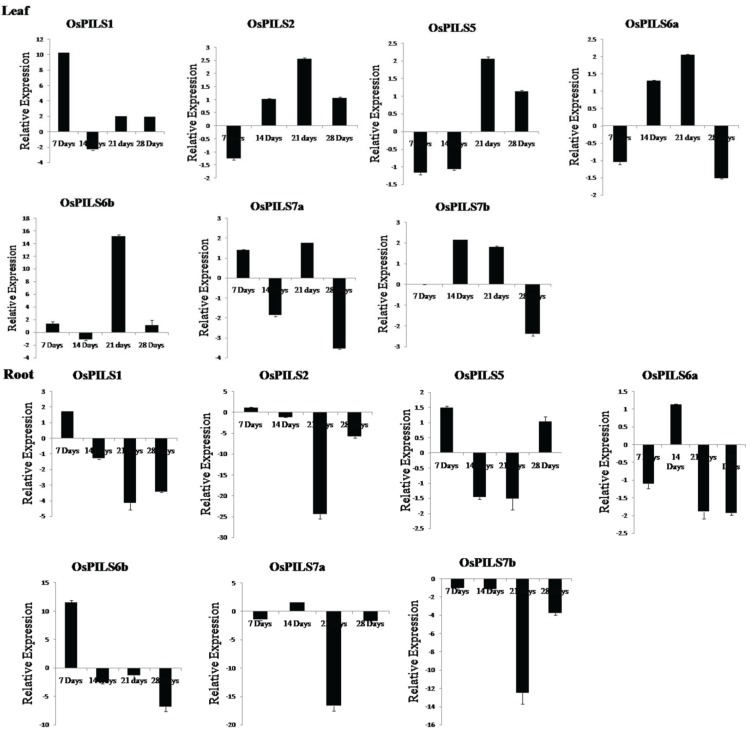
Relative expression of OsPILS genes in leaf and root tissues treated with 5 µM IAA (indole-3-acetic acid). The relative gene expression of OsPILS genes shows that all OsPILS genes were up-regulated in auxin treated leaf tissues and down-regulated in auxin treated root tissues at 21 days.

The *OsPILS1*, *OsPILS6b* and *OsPILS7a* genes were significantly up-regulated, while *OsPILS2*, *OsPILS5* and *OsPILS6a* were significantly down-regulated in seven day old rice plants. The relative expression of *OsPILS7b* was not detected in seven day old plants (Figure 7). In 14 day old plants, *OsPILS2*, *OsPILS6a* and *OsPILS7b* genes were up-regulated and *OsPILS1*, *OsPILS5*, *OsPILS6b* and *OsPILS7a* genes were down regulated. All *OsPILS* genes were observed to be up-regulated by more than two-fold in 21 day old rice plants. In 28 day old plants, *OsPILS1*, *OsPILS2*, *OsPILS5* and *OsPILS6b* genes were up-regulated and *OsPILS6a*, *OsPILS7a* and *OsPILS7b* genes were down regulated (Figure 7).

### 3.4. Expression of OsPILS Genes in Root Tissue Treated with IAA

The *OsPILS1*, *OsPILS2*, *OsPILS5* and *OsPILS6b* genes was significantly up-regulated, while the *OsPILS6a*, *OsPILS7a* and *OsPILS7b* genes were significantly down regulated in seven day old rice seedlings (Figure 7). At the 14-day time point, only *OsPILS6a* and *OsPILS7a* genes were up-regulated, whereas all other *OsPILS* genes were down regulated. At 21 days, all *OsPILS* genes were down regulated. At 28 days, all *OsPILS* genes except *OsPILS5* were down regulated (Figure 7).

### 3.5. Expression of OsPILS Genes in Leaf Tissue Treated with Cytokinin

**Figure 8:**
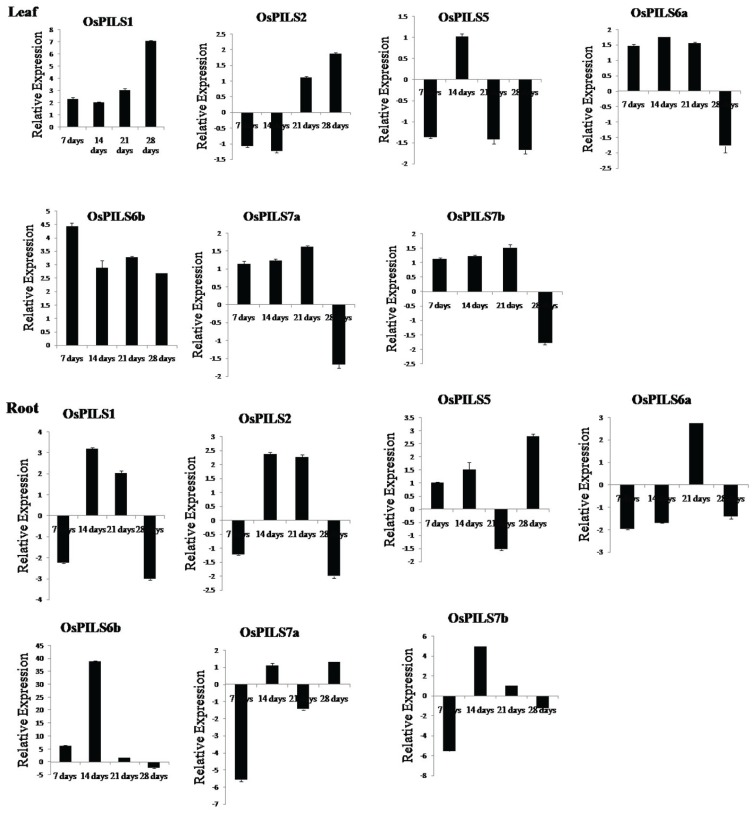
Relative expression of OsPILS genes in leaf and root tissues treated with 5 µM BAP (6-benzylaminopurine).Maximum of OsPILS genes were up-regulated in leaf tissues. Specifically, the OsPILS1 and OsPILS6b genes were up-regulated at seven, 14, and 21 days in cytokinin treated leaves. In cytokinin treated root tissues, the majority of OsPILS was up-regulated at 14 and 21 days.

The *OsPILS1*, *OsPILS6a*, *OsPILS6b*, *OsPILS7a* and *OsPILS7b* genes were up-regulated, while *OsPILS2* and *OsPILS5* genes were down regulated in seven day old cytokinin treated rice seedlings (Figure 8). At 14 days, the *OsPILS1*, *OsPILS5*, *OsPILS6a*, *OsPILS6b*, *OsPILS7a* and *OsPILS7b* genes were up-regulated, but *OsPILS2* was not. At day 21 days, all *OsPILS* genes were up-regulated except for the *OsPILS5* gene (Figure 8). At day 28, the *OsPILS1*, *OsPILS2* and *OsPILS6b* genes were up-regulated, while *OsPILS5*, *OsPILS6a*, *OsPILS7a* and *OsPILS7b* genes were down regulated.

### 3.6. Expression of OsPILS Genes in Root Tissue Treated with Cytokinin

Expression of the *OsPILS5* and *OsPILS6b* genes in cytokinin treated root tissue was up-regulated, while that of the *OsPILS1*, *OsPILS2*, *OsPILS6a*, *OsPILS7a* and *OsPILS7b* genes were down regulated in seven day old rice seedlings (Figure 8). At day 14, all *OsPILS* genes except for *OsPILS6a* wereup-regulated. At day 21, the *OsPILS1*, *OsPILS2*, *OsPILS6a*, *OsPILS6b* and *OsPILS7b* genes wereup-regulated, whereas the *OsPILS5* and *OsPILS7a* genes were down regulated (Figure 8).

## 4. Discussion

Auxin (indole-3-acetic acid), which is one of the most important phytohormones found in plant, possesses a unique position among plant growth regulatory substances [17,36,37,38,39,40,41,42]. Specifically, auxin acts as a prominent signaling molecule via its local accumulation or depletion in selected cells, as well as a spatial and temporal reference for changes in developmental programmes. The temporal and spatial distribution of auxin relies on its metabolism (biosynthesis, conjugation and degradation) and cellular transport [43,44]. Interaction and coordinated auxin transport in plants relies on flexible networks that metabolize auxin in response to several environmental and developmental changes encountered by plants [35,37]. The auxin distribution and its differential accumulation in varying plant tissues are created in response to internal developmental programs or endogenous signaling cascades [38,45,46]. This process is carried out by auxin metabolism and transport. The auxin transport system has been shown to be involved in modulation of plant development, and multiple transporter proteins like PINS and PILS that are required to maintain directional auxin flows within and between different organs and tissues. Thereby, plants accommodated maximum number of auxin transporter proteins like *PINs* and *PILS* genes to broaden its optimum functionalities. This auxin transport is realized over both short and long distance distributions [8,47].

The members of auxin efflux carrier proteins associated with auxin transports have been characterized by their presence as integral membrane proteins [46]. In the present study, all OsPILS proteins were predicted to be membrane localized (Figure 5) and contain putative auxin efflux carrier domains (Figure 3). Each PILS protein possesses a central hydrophilic loop flanked on each side by five transmembrane domains. The auxin efflux carriers (PINs) are divided into long (PIN1, PIN2, PIN4 and PIN7) and short (PIN3, PIN5) efflux carrier proteins depending on the length of their hydrophilic loop [8]. The long efflux carriers primarily show polar plasma membrane localization, provide the directional auxin transport and play major roles in auxin dependent processes in plant development, embryo development, organogenesis and tropism [12]. In contrast to long efflux carriers, short efflux carriers have reduced central hydrophilic loops and do not localize to the plasma membrane [19]. Rather, these carriers localized to the endoplasmic reticulum (AtPIN5), suggesting that they play a role in intracellular auxin distribution and regulation of cellular auxin homeostasis [20]. In the present study, all of the OsPILS proteins were found to possess a short hydrophilic loop and therefore assumed to be endomembrane localized (Table 3). Barbez *et al.* (2012) also reported that all AtPILS were endomembrane localized [10]. In the phylogenetic tree, the short transmembrane domain containing proteins OsPIN5 and AtPIN5 were very close to each other (Figure 6). The sub-cellular localization prediction and phylogenetic analysis provides sufficient information regarding the presence of the short hydrophilic loop of OsPILS proteins. The majority of OsPILS were observed to be sub-cellularly localized either in endoplasmic reticulum or vacuoles (Table 3). Similar results have been reported for AtPILS [10]. All the AtPILS proteins were sub-cellularly localized to the endoplasmic reticulum [10]. In the present study, the presence of OsPILS7b proteins was briefly observed in the plasmamembrane (Table 3). The presence of OsPILS proteins in the endoplasmic reticulum/vacuoles that facilitates auxin accumulation inside the cells contributes to the possibility of compartmentalized regulation of auxin metabolism [10]. Multiple sequence alignment revealed that OsPILS proteins contain N- and C-terminal conserved consensus sequences. The N-terminal conserved consensus sequence was N-x-G-N, while the C-terminal conserved consensus sequences were A-P-L and G-G-N-L. Independent functionalities of these domains have yet to reported; hence, more in depth investigations are required to infer the functionalities of N- and C-terminal conserved consensus sequences. Although the central hydrophilic loops of PILS proteins are highly variable, they still contain conserved threonine amino acids. Threonine amino acid is the likely target phosphorylation site of upstream kinases [48]. The presence of conserved threonine amino acids in all OsPILS genes indicates that their functions are regulated by kinase modulated phosphorylation processes [49].

**Table 3:** Sub-cellular localization of OsPILS proteins. Prediction shows that all OsPILS are integral transmembrane proteins and localized to sub-cellular compartment like endoplasmic reticulum and vacuoles. The OsPILS7b was observed to be localized to endoplasmic reticulum as well as plasmamembrane. Prediction of sub-cellular localization was carried out using online available WOLF PSORT protein sub-cellular localization prediction software (http://cbrc3.cbrc.jp/cbrc/news/wolf_eng.html).

Gene	Protein domains	Putative Subcellular Localization
OsPILS1	Integral membrane protein	Vacuolar
OsPILS2	Integral membrane protein	Endoplasmic reticulum
OsPILS5	Integral membrane protein	Endoplasmic reticulum
OsPILS6a	Integral membrane protein	Vacuolar
OsPILS6b	Integral membrane protein	Endoplasmic reticulum
OsPILS7a	Integral membrane protein	Endoplasmic reticulum
OsPILS7b	Integral membrane protein	Plasmamembrane & ER

In 2012, the presence of *PILS* genes (sub-family of PIN genes) in *Arabidopsis thaliana* was reported for the first time [10]. Barbez *et al.* (2012) reported the presence of seven AtPILS genes in *A. thaliana.* Genome wide identification of *PILS* genes in *O. sativa* led to identification of seven *OsPILS* genes that were considered *PIN*-like genes based on their similar topologies. Although PIN and PILS proteins share similar predicted protein topology, they do not share pronounced protein sequence identity [10]. Accordingly, it is very difficult to identify PILS proteins by conventional BLAST approaches. Interpro scan analysis of all OsPILS proteins revealed the presence of an auxin career domain (Figure 3). PILS proteins are conserved throughout the plant lineage from unicellular algae from *Ostreococcus* and *Chlamydomonas* (where PIN genes are absent) to angiosperms [10], indicating that they are evolutionarily older than PIN proteins. To understand the role of PILS as an auxin carrier, Barbez *et al.* (2012) cultivated PILS2 oestradiol-inducible tobacco BY-2 cells and conducted a ^3^H-IAA accumulation assay. They found that *AtPILS2* induction increased the accumulation of radioactivity in BY-2 cells, indicating that *AtPILS2* carry out auxin transport process. In accordance with the auxin accumulation assay in BY-2 cells, *pils2 pils5* double-mutant protoplast showed a significantly higher level of auxin export, indicating reduced auxin retention capacity in loss of function mutant [10]. The expression of *AtPILS2*, *AtPILS3* and *AtPILS7* in *Saccharomyces cerevisiae* also led to increased retention of exogenously applied auxin.

Barbez *et al.* (2012) also reported that *AtPILS* genes are expressed broadly in different tissue and that *AtPILS2-AtPILS7* was transcriptionally up-regulated by application of auxin. In the present study, *OsPILS* were also up-regulated in auxin treated plants (Figure 7). The major finding of this transcriptome analysis was that all of the *OsPILS* genes were up-regulated at week 3 in auxin treated leaf tissues, while all of the *OsPILS* genes in auxin treated root tissues were down regulated (Figure 7). In cytokinin treated leaf tissues, with the exception of *OsPILS5*, all other *OsPILS* genes were up-regulated at week 3 (Figure 8). Similarly, with the exception of *OsPILS5* and *OsPILS7a*, all other *OsPILS* genes were up-regulated in cytokinin treated root tissue at week 3 (Figure 8). The exceptional differential gene expression of the *OsPILS5* gene may be due to its different topology in transmembrane domain with other *OsPILS* genes (Figure 5). The maximum up-regulation of *OsPILS* genes at week 3 reflects their significant roles in growth and development during this time period. This study also revealed some important aspects of *O. sativa* plant development at week 3, and indicated that major hormonal and developmental changes might have occurred during this time period to shape the plant for its future development. The *OsPILS1* gene was up-regulated in auxin treated leaf tissues in the first, third and fourth week (Figure 7), while it was down regulated in the second, third, and fourth week in auxin treated root tissues (Figure 7). The *OsPILS2* gene was consistently up-regulated in the second, third, and fourth week in auxin treated leaf tissues, while it was down regulated in the second, third and fourth week in auxin treated root tissues (Figure 7). These findings suggest that application of exogenous auxin reversed expression of the respective *OsPILS* genes in root tissues. *OsPILS6a* was up-regulated in the second week in auxin treated leaf and root tissues (Figure 7). However, in the majority of cases, *OsPILS6a* was down regulated in auxin treated root tissues in the first, third and fourth week. The up-regulation of *OsPILS6a* in auxin treated leaf and root tissues suggests its important role during the second week. The expression of *OsPILS6b* was up-regulated except during the second week in auxin treated leaf tissues, while it was down regulated at week 2, 3 and 4 in root tissues (Figure 7). The application of exogenous auxin reversed the expression of *OsPILS* genes in root tissues. In the case of *OsPILS7b*, all genes were down regulated in auxin treated leaf tissues at all time points (Figure 7), suggesting that exogenous application of auxin has significant effects on *OsPILS7b* gene expression in the root tissue. At week 4, with the exception of *OsPILS5*, all other *OsPILS* genes were down regulated in auxin treated root tissues (Figure 7). Overall, the differential expression of *OsPILS* genes in response to exogenous auxin treatment indicates their roles in auxin-regulated processes in *O. sativa*.

In cytokinin treated leaf tissues, the *OsPILS1* and *OsPILS6b* genes were up-regulated in all four time course experiments (Figure 8). Similarly, the *OsPILS6a*, *OsPILS7a* and *OsPILS7b* genes were up-regulated in the first, second and third week, suggesting their important roles in cytokinin treated leaf tissue (Figure 8). In the third week, all genes except *OsPILS5* were up-regulated in cytokinin treated leaf tissues. The significant differential expression of the *OsPILS5* gene may be due to the absence of a proper transmembrane domain and the presence of a slightly dissimilar topology relative to other OsPILS proteins (Figure 5). Similar trends in expression of the *OsPILS5* gene were observed in auxin treated leaf tissue during the same time period when compared to other *OsPILS* genes. The up-regulation of *OsPILS* genes in leaf tissue in both auxin and cytokinin treated plants during week 3 shows its significant roles in the plant developmental process during these time periods. Among the seven *OsPILS* genes, all except *OsPILS5* and *OsPILS7a* were up-regulated in cytokinin treated root tissue at week 3 (Figure 8). This finding is opposite to the gene expression pattern observed in auxin treated root tissue, where all genes were down regulated (Figure 7). In the fourth week, all *OsPILS* genes except *OsPILS5* were down regulated in root tissues, regardless of auxin and cytokinin treatment.

Barbez *et al.*, (2012) reported that the *AtPILS2* and *AtPILS5* genes showed overlapping expression in the root meristematic zone, suggesting that they had a redundant role in regulation of root growth, which was also found to be true for the *OsPILS2* and *OsPILS5* genes. Both of these genes were up-regulated in root tissue in the early phase (first week) of plant development, suggesting their crucial roles in root development. *AtPILS5* gain of function mutant reduced lateral rooting, and the expression of *AtPILS1*, *AtPILS3* and *AtPILS5* under root hair specific promoter following auxin treatment led to repressed root hair growth. This may have been due to the PILS-dependent regulation of auxin signaling and homeostasis.

## 5. Conclusions

In this study, genome wide identification of *OsPILS* genes revealed a distinct protein family that regulates intracellular auxin transport and homeostasis. The results highlighted the developmental and evolutionary importance of *OsPILS* in auxin regulation. However, more in depth investigations are necessary to understand the potential interplay or possible diversified function of endoplasmic reticulum localized *OsPILS* genes and *AtPILS* genes in plants. High throughput mutant analysis of *OsPILS* genes of rice will help elucidate their roles in root development.

## Supplementary Files

Supplementary File 1
