# Supplementary material for: Identification and Expression Analysis of PIN-Like (PILS) Gene Family of Rice Treated with Auxin and Cytokinin
Source: Genes (Basel). 2015 Jul 16;6(3):622–40. doi: 10.3390/genes6030622 (PMC4584321; doi:10.3390/genes6030622)
Supplement: Supplementary File 1 [file genes-06-00622-s001.pdf]

Supplementary Information

Supplementary Data File 1

Multiple sequence alignment of OsPIN, AtPIN and OsPILS protein. Sequence alignment shows, alignment of OsPILS starts towards the end of the OsPIN and AtPIN sequence. This shows that, OsPILS and PIN proteins are highly dissimilar in their sequence.

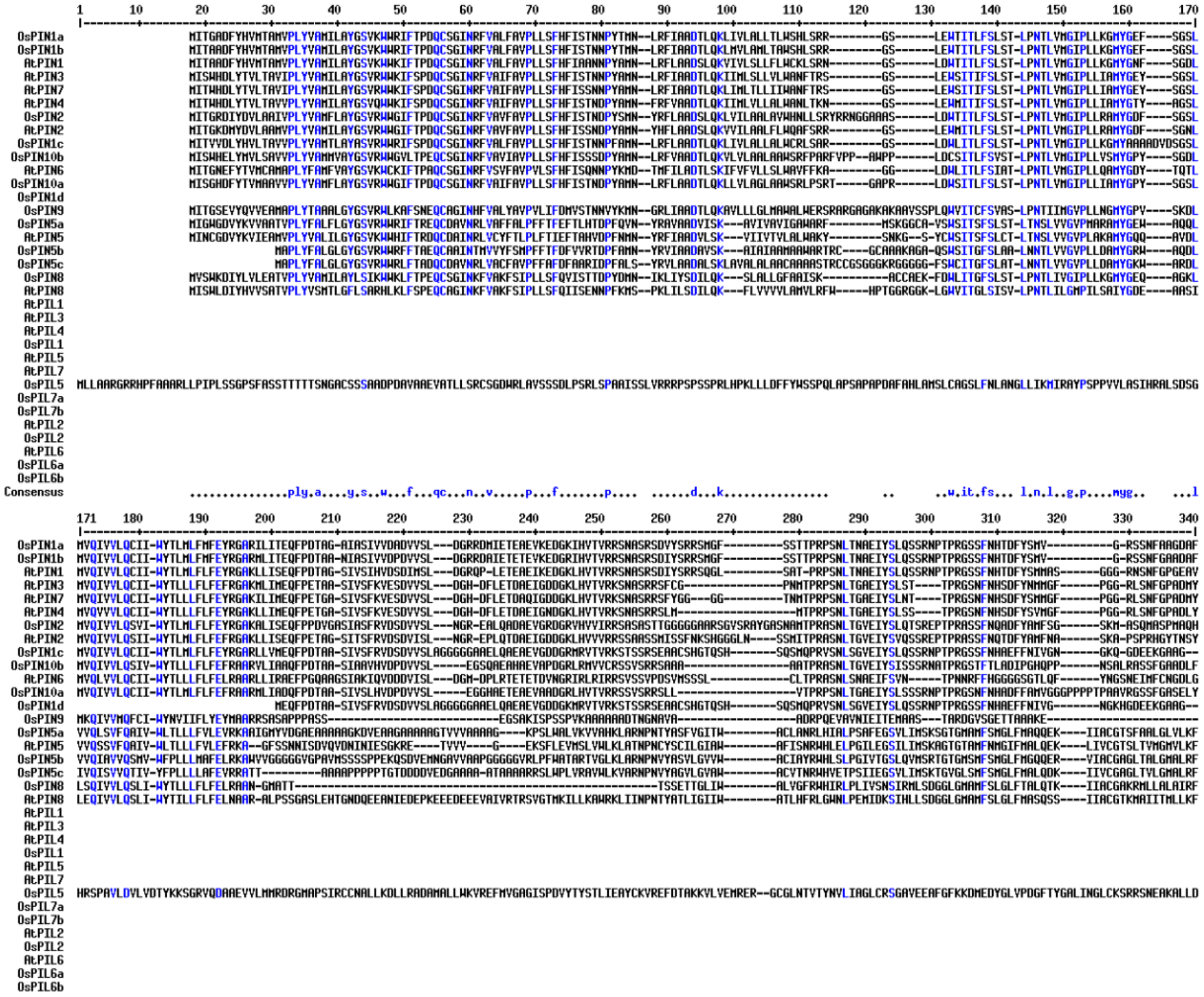

S2

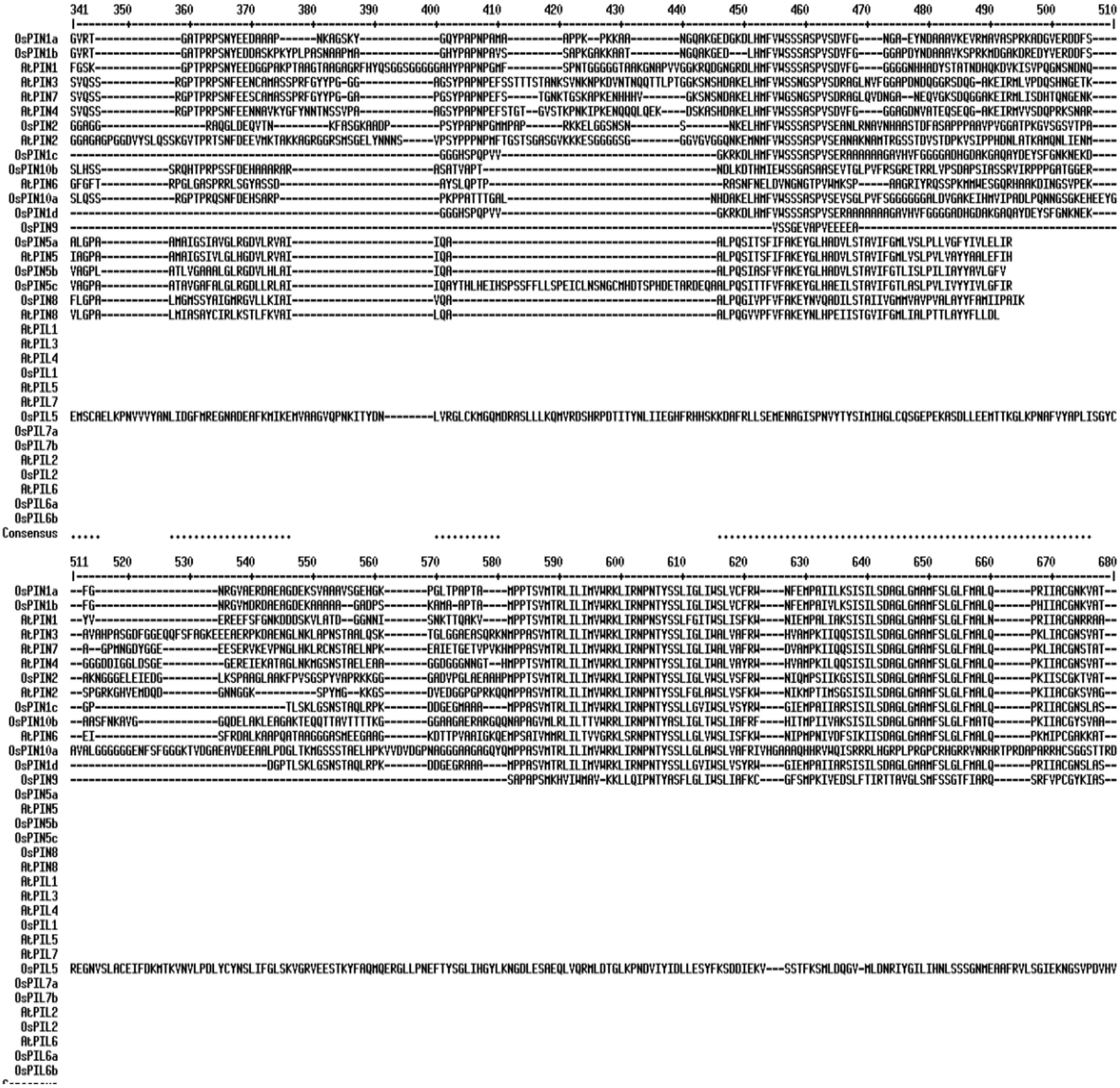

```
           681  690       700       710       720       730       740       750       760       770       780       790       800       810       820       830       840       850
           |--------+---------+---------+---------+---------+---------+---------+---------+---------+---------+---------+---------+---------+---------+---------+---------+---------|
  OsPIN1a  --FAMAVRFLTGPAVMAAASIAVGLRGTLLHVAIVQAALPQGIVPF----VFAKEYSVHPDILSTAVIFGMLIALPITLVYYILLGL
  OsPIN1b  --YAMAVRFLAGPAVMAAASFAVGLRGTLLHVAIVQAALPQGIVPF----VFAKEYSVHPSILSTAVIFGMLIALPITLVYYILLGL
   AtPIN1  --FAAAMRFVVGPAVMLVASYAVGLRGVLLHVAIIQAALPQGIVPF----VFAKEYNVHPDILSTAVIFGMLIALPITLLYYILLGL
   AtPIN3  --FAMAVRFLTGPAVMAVAAIAIGLRGDLLRVAIVQAALPQGIVPF----VFAKEYNVHPAILSTGVIFGMLIALPITLVYYILLGL
   AtPIN7  --FAMAVRFFTGPAVMAVAAMAIGLRGDLLRVAIVQAALPQGIVPF----VFAKEYNVHPAILSTGVIFGMLIALPITLVYYILLGL
   AtPIN4  --FAMAVRFITGPAIMAVAGIAIGLHGDLLRIAIVQAALPQGIVPF----VFAKEYNVHPTILSTGVIFGMLIALPITLVYYILLGL
   OsPIN2  --FAMAVRFLTGPAVIAATSIAIGLRGVLLHVAIVQAALPQGIVPF----VFAKEYNCHPQILSTAVIFGMLIALPITILYYVLLGI
   AtPIN2  --FAMAVRFLTGPAVIAATSIAIGIRGDLLHIAIVQAALPQGIVPF----VFAKEYNVHPDILSTAVIFGMLVALPVTVLYYVLLGL
  OsPIN1c  --YAMAVRFLVGPAVMAAASIAVGLRGVLLHIAIVQAALPQGIVPF----VFAKEYNVHPNILSTAVIFGMLIALPITLVYYILLGL
 OsPIN10b  --ASMGVRFFFGPAIMAAASAAVGIRGTLLRIAIVQAALPQGIVPF----VFAKEYNLHATILCTLVIFGMLIALPITLVYYIILGLL
   AtPIN6  --MGMLIRFISGPLFMAGASLLVGLRGSRLHAAIVQAALPQGIVPF----VFAREYNLHPDLLSTLVIFGMIVSLPVTILYYVLLGL
 OsPIN10a  CAFCFCKRIQCPPGHPEHSGNFWHANSSSNHIAVLHPSWTMIKKAYGRSHIKRKKWGQRERKKSDPVHLKQRMLICIACCRTLPMMTRQRSCYRAIYLCDICTRDNDRMYSNITDIRYVFC
  OsPIN1d  --YAMAVRFLVGPAVMAAASIAVGLRGVLLHIAIVQAALPQGIVPF----VFAKEYNVHPNILSTAVIFGMLIALPITLVYYILLGL
   OsPIN9  --FSMVIKFLIGPVVMLFASLVIGMHGTLLHIAVVQAALPLAVTSF----VYAEEYKVHADIMSTGVILGIFISLPVTIVYYILLGL
  OsPIN5a
   AtPIN5
  OsPIN5b
  OsPIN5c
   OsPIN8
   AtPIN8
   AtPIL1                                                                                    MSLTQSAKLHIIHQSIGKITTRLYDTSPFHGDSHCKQR-RVKDNCIQFNMNISNVLKCYSEIESFSINQRIRRSISLFLSILRSF---------YSMRMRLL
   AtPIL3                                                                                                                                                                                     MVKLL
   AtPIL4                                                                                                                                                                                      MKLL
   OsPIL1                                                                                                                                                                                      MGLL
   AtPIL5                                                                                                                                                                                      MGFW
   AtPIL7                                                                                                                                                                                      MGFL
   OsPIL5  YSSLISGLCKTADREKAFGILDEMSKKGVDPNIVCYNALIDGLCKSG---DISYARNVFNSILAKGLVPNCVTYTSLIDGSCKVGDISNAFYLYNEMLATGITPDA-FVYSVLTTGCSSAGDLEQAMFLIEEMFL--RGHASISSFNNLVDGFCKRGKMQETLKLLHVIM
  OsPIL7a                                                                                                                                                                                      MGFL
  OsPIL7b                                                                                                                                                                                      MGFL
   AtPIL2                                                                                                                                                                           MSGFSSG-NVNSRVV--
   OsPIL2                                                                                                                                                                           MDWLSANLQVNPQVTVH
   AtPIL6                                                                                                                                                                   MIARILAALADSMEMPVAAGGGSVL
  OsPIL6a                                                                                                                                                                  MMERSLMEALATAAQGG--TVGTSVF
  OsPIL6b                                                                                                                                                                  MMGRSVLEMVVAAAQGGGGAAGESVL
Consensus    ........................................    ....................................                                                                                              ....

           851  860       870       880       890       900       910       920       930       940       950       960       970       980       990       1000      1010      1020
           |--------+---------+---------+---------+---------+---------+---------+---------+---------+---------+---------+---------+---------+---------+---------+---------+---------|
  OsPIN1a
  OsPIN1b
   AtPIN1
   AtPIN3
   AtPIN7
   AtPIN4
   OsPIN2
   AtPIN2
  OsPIN1c
 OsPIN10b
   AtPIN6
 OsPIN10a
  OsPIN1d
   OsPIN9
  OsPIN5a
   AtPIN5
  OsPIN5b
  OsPIN5c
   OsPIN8
   AtPIN8
   AtPIL1  DLFITSSIPVAKILLITGIGFYLALDQVNILNHDARKQLNNIV--FYVFSPSLVASSLSETITYESMVKMWFMPLNVLLTFIIGSFLGWIVIKITKPPSHLRGIIVGCCAAGNLGNMPLIIIPAICNEKGSPFGDPES-CEKFGLG-YIALSMAIGAIYIWTY-------
   AtPIL3  ELFITSSKPVVEILLITSVGFYMALDGVNLLGHDARKYLNNIV--FYVFSPSLIGSRLADSVTYESLVKMWFMPVNVLLTFIIGSLLGWIVIVITKPPSHLRGLILGCCAAGNLGNMPLIIIPAVCKEKGGPFGDPES-CQKYGMG-YVALSMAMGSIYIWTY-------
   AtPIL4  ELFIASSKPVVETLLITSVGFYLALDTVNLLGHDARKHLNNIV--FYVFSPSLIGSRLADSVTYESLVKMWFMPVNVLLTFMIGSLLGWIVIVITKPPSQLRGLIISCCASGNLGTMPLIIIPAICKEKGGPFGDSES-CEKYGMG-YVTLSMTAFFISVYKHDTNWYVS
   OsPIL1  ELFITACVPVLNMLLVTGVGSFLATDFVGILNKDARKYLNNIV--FYVFNPSLVATYLAQTITLEGLAKLWFMPVNILLACTFGLILGWIVVHVTRAPARLRGLILGCCSAGNWGNIFLIIIPALCKEKGSPFGAPDV-CHTYGLA-YSSLSMALGAVFLWTVAYNIMRA
   AtPIL5  SLLEVASMPVIQVLFMSLVGAFMASDRCKLFPVEARNSMNKVV--FVLFAPALMFANLAQTVTLEDIISWWFMPVNMGLTFLIGGLLGWLVVKILKPPPYLEGLIVATCSAGNMGNLPIILVPAICDEDKSPFGN-RSVCRTVGLS-YASFSMALGGFYIWTYTFRLIKG
   AtPIL7  ELLEVASMPIVQVLLISVLGAFLATDYCSLLSADTRRSVNKLV--FVVFTPCIMFANLAETVTLQDIISWWFMPINVGITFLVGGILGWLVVKLLNPKPQLHGLIIATCASGNMGNLMLILVPAICDEEGSPFGN-RSVCRSIGLS-YASFSMALGGFYIWTYSYQLVRS
   OsPIL5  GRGLVPNALTIENIISGLSEAGKLSEVHTIFVELQQKTSESAARHFSSLFMDMINQGKIPLDVVDDMIRDHCKEGNLDKALMLRDVI--VAKSAPMGCSSYLAIVDNLCRKGKLSEALNLLKEM--DKRGNLQPTLVALLGIFWFRRHHHILNKLGGLYIWTHTYSLMKK
  OsPIL7a  SLLLVASMPIVQVLLIGVIGAFLASGYSKILTSSALRDMNKVV--FTVFTPSLMFASLAKTVTLSDVISWWFMPVNIGITFIVGGTLGWIACKILKPPQHFRGMIIAFCSAGNLGNLLLIIVPAVCDEDGNPFGKDRSLCRSRGLS-YSSLSMALGGLFIWTHTYSLMQK
  OsPIL7b  ALLLVASMPVVQVLLIGVVGAFLASGYSNILTSSALSDMNKVV--FTVFTPSLMFASLARTVTFSDVISWWFMPINIGITFMAGGTLGWIACRILKPPQHFRGMIIAFCSAGNLGNLLLIVVPAVCDEDGNPFGKDSSRCRSLGLS-YSSLSMALGGLYIWTHTYSLMKK
   AtPIL2  -DILSGVVPLLKLICLTVIGLLLAHPKTQLVPRATFRLLSKLV--FALFLPCLIFTELGESITLDNIVQWWFIPVNVLLSAVVGSLIGYLVVLICRPPPEFNRFTIVMTAFGNTGNLLLAIVSSVCHTKTNPFGPN---CNSRGVS-YVSFAQWVAVILVYTVVYHMMEP
   OsPIL2  GDWVSAVMPLMKLLCLTVIGLVLSNPRTQIIPKATFKLLSKLV--FALFLPCLIFVHLGQSVTIQNILDWWFIPVNVLISTAIGCILGYIVALICRPPPQFFRFTVIMTGFGNTGNLPIAIIGSVCHTTDHPFGPG---CHRKGVA-YVSFAQWVAVILVYTLVYHMMEP
   AtPIL6  GTIKIAVMPIAKVFTMCFLGLLMASKYVNILPPSGRKLLNGLV--FSLLLPCLIFSQLGQAVTLQKMLQWWFIPVNVVLGTISGSIIGFIVASIVRPPYPYFKFTIIQIGVGNIGNVPLVLLAALCRDTSNPFGDSEK-CSIDGTA-YISFGQWVGAIILYTYVYQMFAP
  OsPIL6a  DMLKYAVLPIAKVFTVCFMGFLMASKYVNILQPNGRKLLNGLV--FSLLLPCLIFSQLGRAITIEKMLQWWYIPVNIVVGAVSGSLIGFVVASIIRPPYPYFKFTVIHIGIGNIGNIPLVLIAALCRDPTNPFGDSDK-CNEDGNA-YISFGQWVGAIIVYTYVFKMLAP
  OsPIL6b  GMFRYAVLPIAKVFVVCFMGFLMASKRVGVLKPSGRKLLNALV--FSLLLPCLIFAQLGRSITIDKIMEWWFIPANIALGAVSASLVGLIVALIVRPPYPYFKFTITHIGIGNIGNIPLVLISALCRDQLNPFGDSNK-CTQDGNA-YLSFGQWVGAIIVYTYVFKMLAP
```

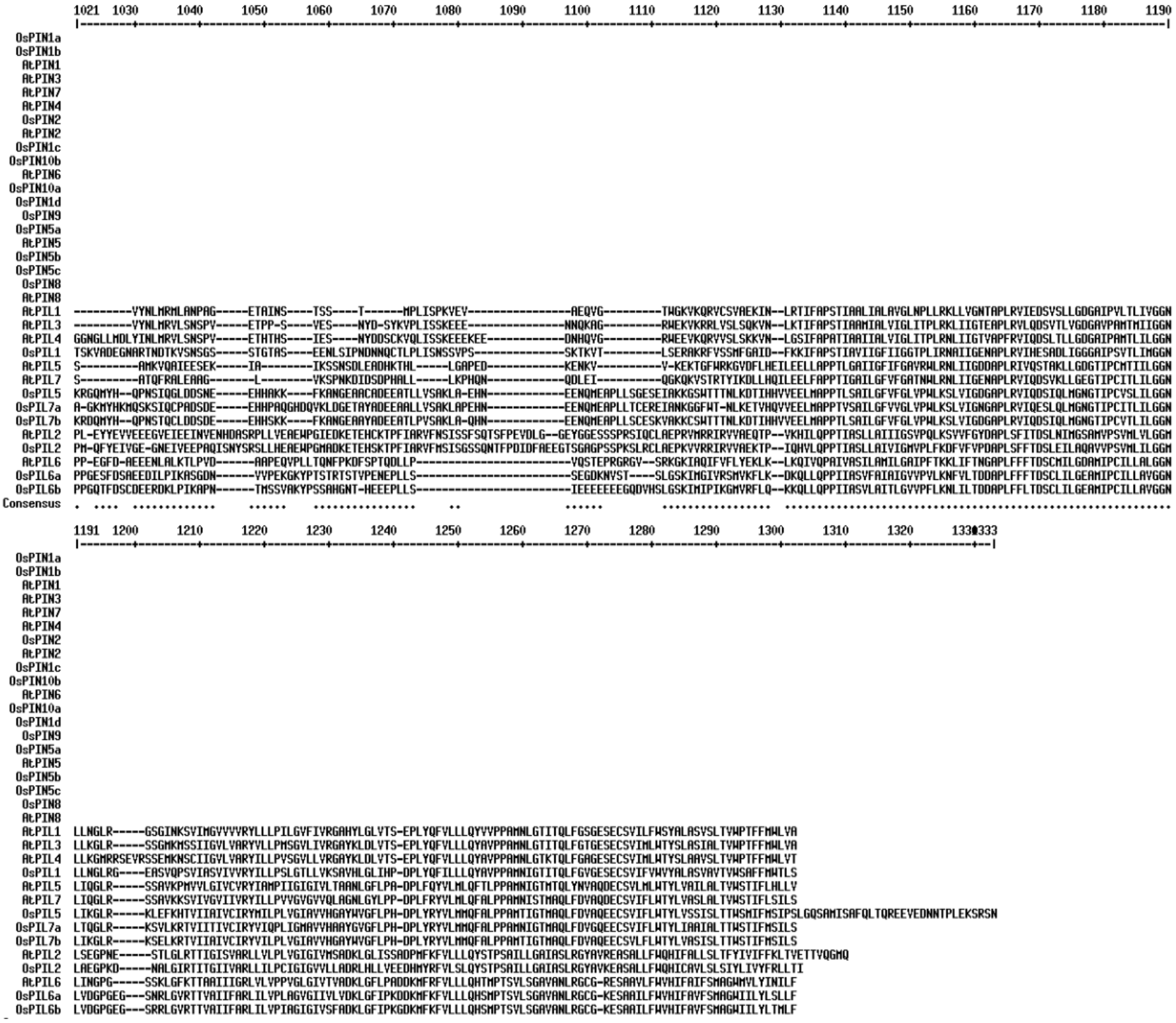

© 2015 by the authors; licensee MDPI, Basel, Switzerland. This article is an open access article distributed under the terms and conditions of the Creative Commons Attribution license (http://creativecommons.org/licenses/by/4.0/).
